# Vasoplegic syndrome following cardiothoracic surgery—review of pathophysiology and update of treatment options

**DOI:** 10.1186/s13054-020-2743-8

**Published:** 2020-02-04

**Authors:** Laurence W. Busse, Nicholas Barker, Christopher Petersen

**Affiliations:** 1 Department of Medicine, Emory University, Emory Critical Care Center, Atlanta, GA USA; 2 Emory Johns Creek Hospital, 6325 Hospital Parkway, Johns Creek, GA 30097 USA; 3 Department of Pharmacy, Emory St. Joseph’s Hospital, Atlanta, GA USA; 4 Department of Pharmacy, Stanford Health Care, Palo Alto, California, USA

**Keywords:** Vasoplegic syndrome, Shock, De-catecholaminization, Hydroxocobalamin, Angiotensin II, Cardiopulmonary bypass

## Abstract

Vasoplegic syndrome is a common occurrence following cardiothoracic surgery and is characterized as a high-output shock state with poor systemic vascular resistance. The pathophysiology is complex and includes dysregulation of vasodilatory and vasoconstrictive properties of smooth vascular muscle cells. Specific bypass machine and patient factors play key roles in occurrence. Research into treatment of this syndrome is limited and extrapolated primarily from that pertaining to septic shock, but is evolving with the expanded use of catecholamine-sparing agents. Recent reports demonstrate potential benefit in novel treatment options, but large clinical trials are needed to confirm.

## Background

Vasoplegic syndrome, a form of vasodilatory shock following cardiopulmonary bypass (CPB), may affect up to half of all patients undergoing major cardiovascular surgery [1–6]. The pathophysiology of vasoplegic syndrome is similar to that of sepsis. A large number of patients require vasopressors post-operatively to maintain adequate tissue perfusion. The need for escalating vasopressors is associated with a higher incidence of morbidity and mortality. Research describing the use of agents in refractory vasoplegic syndrome is limited primarily to case series and case reports. The present review discusses the pathophysiology of vasoplegic syndrome and evaluates the various treatment options with insight from personal experience with novel non-catecholamine therapies.

Vasoplegia is characterized by a normal or augmented cardiac output with low systemic vascular resistance (SVR) causing organ hypoperfusion. The exact definition has varied but typically is considered when shock occurs within 24 h of CPB in the setting of a cardiac index (CI) is greater than 2.2 L/kg/m^2^ and SVR less than 800 dyne s/cm^5^. These criteria are relatively non-specific and found in other disease states such as sepsis, adrenal insufficiency, and hepatic failure, among others, with the distinction being the etiology of the shock (infection in the case of sepsis and exposure to extracorporeal circulation in the case of vasoplegia) [7]. Treatment of this syndrome is usually limited to the initiation of vasopressors to maintain adequate perfusion pressures via the targeting of a specific mean arterial pressure (MAP). Due to the similarity in between vasoplegic syndrome and sepsis, along with paucity in supporting evidence, many of the treatment options used in septic shock have been extrapolated to use in vasoplegic syndrome.

Vasoplegic syndrome following cardiovascular surgery accounts for less than 5% of all circulatory shock [8]. Despite this, between 5 and 50% of patients undergoing cardiac surgery may experience vasoplegic syndrome with high morbidity and mortality rates in those patients [9, 10]. Incidence is higher in patients with preoperative risk factors including preoperative use of antihypertensive medications, a large number of comorbidities, warmer core temperatures while on bypass, and a longer duration on bypass [10].

## Pathophysiology

**Fig. 1:**
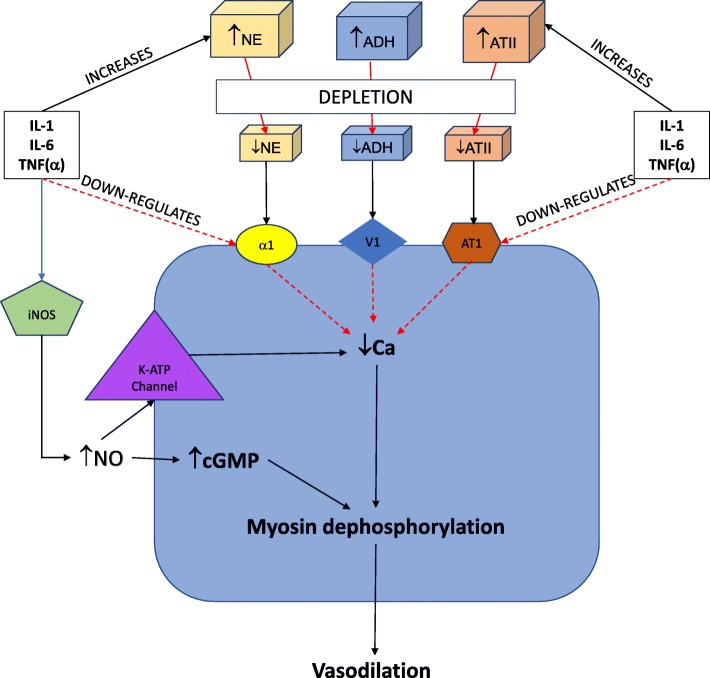
Pathophysiology of vasoplegia. Physiologic contraction of vascular smooth muscle occurs in response to intracellular calcium, which cause myosin phosphorylation leading to myosin-actin filament crosslinking and vasoconstriction. Cytoplasmic calcium is increased through alpha-1 adrenergic receptor, vasopressin-1 receptor, and angiotensin type-1 receptor activation. Inflammatory mediators released during cardiopulmonary bypass can lead to adrenoreceptor desensitization, an immediate increase in vasoconstrictive mediators with subsequent depletion, and the production of nitric oxide (NO). NO leads to an increase in cGMP, which inhibits calcium into cells, leading to muscle relaxation. NO also activates ATP-sensitive potassium channels (KATP), leading to hyperpolarization and inhibited vasoconstriction

The mechanism by which CPB leads to vasoplegia is multifactorial and depends on several patient characteristics as well as the nature of the surgical procedure. A simplified schematic of the pathophysiology of vasoplegia is presented as Fig. 1. In healthy humans, contraction of vascular smooth muscle occurs as a response to rising levels of intracellular calcium. Increased levels of intracellular calcium cause a cascade of events starting with myosin phosphorylation leading to myosin-actin filament crosslinking and vasoconstriction. The influx of cytoplasmic calcium is generated by agonism of G-protein coupled receptors via catecholamines (alpha-1 adrenergic receptor), arginine vasopressin (vasopressin-1 receptor), and angiotensin II (angiotensin type-1 receptor) [11]. This mechanism is dysregulated during CPB, as the exposure of blood to foreign surfaces inside of the CPB circuit stimulates the release of inflammatory mediators, such as interleukin-1 (IL-1), interleukin-6 (IL-6), and tumor necrosis factor-alpha (TNF). These cytokines stimulate the locus coeruleus and the hypothalamic pituitary-adrenal axis in the paraventricular nucleus which over time leads to adrenoreceptor desensitization and a proinflammatory state [11]. These inflammatory mediators can also potentiate the production of nitric oxide (NO), which is vasodilatory, and in excess, can result in vasoplegic shock. Consequently, norepinephrine is released from sympathetic nerves located in lymphoid organs, epinephrine and cortisol are released from the adrenal cortex, arginine vasopressin (AVP) is released from the hypothalamic axis, and angiotensin II is upregulated as part of the renin-angiotensin-aldosterone axis [12]. As shock persists, there is subsequent depletion of these hormones. This has been elucidated with AVP specifically [13–15]. Landry et al. found that endogenous vasopressin acutely increases in a hypotensive state followed by decreasing concentrations leading to relative AVP deficiency [13]. AVP is of particular importance in vasoplegic syndrome, due to its ability to neutralize the effects of NO and decrease NO production [6].

NO is produced from l-arginine by nitric oxide synthase (NOS) enzymes. NOS enzymes are differentiated based on location and levels of activity. Constitutive endothelial NOS (eNOS) provides a baseline constant production of NO in endothelial cells which can rapidly diffuse into vascular smooth muscle cells and exert its effects. Inducible NOS (iNOS) is stimulated via inflammatory cytokines and can lead to significantly higher levels of NO compared to eNOS, up to 1000-fold [11, 16]. NO increases vasodilation via multiple methods. It activates guanalyl cyclase, an enzyme found in the vascular smooth muscle that catalyzes the dephosphorylation of guanosine triphosphate to cyclic guanosine monophosphate (cGMP), by binding to the heme moiety of the enzyme. cGMP inhibits calcium entry via voltage-gated channels and activates cGMP-dependent protein kinases leading to dephosphorylation of myosin light chains, leading to muscle relaxation [7]. NO also activates ATP-sensitive potassium channels (*K*_ATP_), which creates a hyperpolarized state [15]. In a hyperpolarized state, the secondary intracellular cascade leading to vasoconstriction is inhibited despite activation of G-protein coupled receptors.

CPB-induced inflammatory mediators stimulate iNOS and cause increased release of NO, leading to profound vasodilation [17–19]. This inflammatory response is similar to the systemic inflammatory response syndrome (SIRS) that occurs most notably in sepsis and is further compounded by the innate inflammatory response to surgical trauma, yielding increased inflammation and subsequent loss of vascular tone [18]. As CPB continues, a secondary immune response occurs as a result of the reinfusion of blood from the thoracic cavity through the CPB circuit [5]. A combination of hemolyzed red blood cells, platelets, and recirculated denatured proteins stimulates a secondary immune response. When non-pulsatile CPB is discontinued and pulsatile tissue reperfusion re-occurs, a microvascular ischemia-reperfusion injury leads to further NO production and vasodilation [5, 6].

In severe cases of vasoplegia, norepinephrine responsiveness may be blunted by a number of mechanisms. Adrenergic receptors become phosphorylated inhibiting binding of catecholamines, and increased production of NO interferes with adrenergic receptor activation [11]. These factors in combination with AVP deficiency, acidosis, and cell membrane hyperpolarization secondary to *K*_ATP_ channel stimulation all contribute to the vasoplegic state.

Patient- and treatment-specific factors also contribute to the development of vasoplegia following CPB. Argenziano et al. examined 145 patients undergoing CPB and found that left ventricular ejection fraction (LVEF) < 35% was independently associated with the development of post-CPB vasodilatory shock [20]. This correlation may be due in part to a sustained inflammatory state caused by chronic tissue hypoperfusion and ischemia that is common in patients with heart failure with reduced LVEF. The authors also found the use of angiotensin-converting enzyme (ACE) inhibitors prior to CPB to be an independent predictor of post-CPB vasodilatory shock [20]. ACE inhibitors are commonly used medications in this patient population, and other common medications include beta blockers and calcium channel blockers, which may also be associated with increased risk of vasoplegia [4, 20–22]. The use of vasodilatory inotropes such as dobutamine or milrinone, common both before and after cardiac surgery, may contribute to vasoplegic shock [23]. In addition, patients receiving vasopressors prior to cardiac surgery are also at increased risk [4].

CPB circulatory strategies may contribute to the vasoplegic state. Recirculated blood intraoperatively is associated with increased inflammatory mediators [24]. Cytokine release can occur as a result of hemolysis and the release of free hemoglobin and can be minimized using blood purification techniques [25]. Blood washing can be accomplished using a number of mechanisms, including hemodialysis, adsorption, absorption, and anti-body-mediated removal and have been previously described [26, 27]. Some of these technologies (e.g., CytoSorb®, CytoSorbents Corporation, New Jersey, USA) are not universally available. Circuit characteristics may also mitigate or exacerbate vasoplegia, and strategies such as smaller circuit size or biocompatible circuit coatings have been postulated as ways to reduce inflammation. Reduced circuit exposure using minimal extracorporeal circulation (MECC) was compared to conventional extracorporeal circulation in a meta-analysis of 24 studies and found to be associated with improved short-term outcomes [28]. However, a 2014 systematic review found that only 3 of 8 studies evaluating MECC cited a clinical benefit [29]. Coated circuits mimic the body’s innate endothelial surface via different types of biocompatible molecules (e.g., heparin, poly2-methoxyethylacrylate) and are hypothesized to reduce cytokine activation resulting from blood cell contact with the circuit surface. There is some evidence that supports the use of biocompatible circuitry. A systematic review by Ranucci et al. concluded that coated circuits were associated with a lower rate of transfusion, atrial fibrillation, and a shorter ICU length of stay [30]. A more recent review noted inflammatory suppression in 12 studies, with six reporting a clinical benefit [29]. Despite this, in clinical practice most bypass circuits are not heparin bonded.

## Treatment

Non-mechanical (i.e., blood purification or CPB circuit) treatment options for vasoplegic syndrome include elements of the sympathetic nervous system (catecholamines), the arginine-vasopressin system (vasopressin), and the renin-angiotensin-aldosterone system (angiotensin II), as well as moderators of NO and/or inflammation (methylene blue, hydroxocobalamin, vitamin C, thiamine and corticosteroids). A comprehensive list of all of these agents is presented as Table 1.

**Table 1:** Options for the treatment of vasoplegia

Agent	MOA	Dose
Norepinephrine	Significant α1, α2 agonism Moderate β1 agonism	0.01–3 μg/kg/min
Epinephrine	Significant α1, α2 agonism Significant β1 agonism	0.01–1 μg/kg/min
Phenylephrine	Significant α1, α2 agonism No effect on β1	0.1–5 μg/kg/min
Dopamine	Dose dependent adrenergic agonism α1 agonism as dose increases	1–20 μg/kg/min
Vasopressin	Repletion of vasopressin in ADH depleted state V1 agonism	0.01–0.1 U/min
Vitamin C Thiamine Hydrocortisone	Cofactor for catecholamine synthesis Cofactor of lactate dehydrogenase (increase in lactate clearance) Aids in vitamin C metabolism Repletion of glucocorticoid and mineralocorticoid activity in cortisol depleted state Inhibition of pro-inflammatory cytokines	1.5 g every 6 h 100 mg every 6 h 50 mg every 6 h
Methylene blue	Inhibition of guanylyl cyclase and inducible endothelial NO synthase	1–2 mg/kg
Hydroxocobalamin	Inhibition of NO directly and inducible endothelial NO synthase Inhibition of hydrogen sulfide	5 g
Angiotensin II	AT1 agonism Stimulation of aldosterone release Increase in ADH synthesis	10–40 ng/kg/min

### Catecholamines

Catecholamines (specifically norepinephrine) have long been considered first-line therapy in shock. Data supporting the use of these agents is primarily focused on the septic shock population, with limited evaluation in the vasoplegic population. Norepinephrine and epinephrine agonize the alpha-1 receptor located on vascular smooth muscle resulting in an influx of calcium and subsequently vascular contraction. Compared to norepinephrine, epinephrine has a more potent agonistic effect on the beta-1 receptor which may increase inotropy but may also increase the risk of tachycardia, which is not desirable in patients following cardiac surgery. With regard to the optimal catecholamine agent for the vasoplegia population, norepinephrine may be most tolerated. Norepinephrine has been shown to be less arrhythmogenic than dopamine [31]. Epinephrine, conversely, may worsen heart rate, myocardial oxygen consumption, and the likelihood of arrhythmia, and phenylephrine may worsen systolodiastolic function and ventriculoarterial coupling [32]. A 2006 systematic review that compared multiple agents for treatment of vasoplegia including norepinephrine, dopamine, and phenylephrine concluded that no particular vasopressor was superior to any other, but did recommend that a second agent with a different mechanism of action should be used if blood pressure goals could not be achieved [33]. The use of norepinephrine specifically for vasoplegia was evaluated as part of the Vasoplegic Shock after Cardiac Surgery (VANCS) trial, which compared norepinephrine to vasopressin as first-line therapy in patients recovering from cardiac surgery [34]. In this trial, the primary endpoint of a composite of mortality or severe complications (stroke, requirement for mechanical ventilation for longer than 48 h, deep sternal wound infection, reoperation, or acute renal failure) within 30 days was more likely in the norepinephrine group 49% vs. 32%, unadjusted hazard ratio 0.55; 95% CI 0.38 to 0.80; *p* = 0.0014). However, norepinephrine did not differ from vasopressin with respect to mortality, and the differences in this endpoint were driven by more arrhythmia and acute kidney injury specifically. Be this as it may, higher doses of any catecholamine may be associated with immunosuppression, an increase in myocardial oxygen demand, interference with cellular energy metabolism, oxidative stress, arrhythmias, and risk of necrosis secondary to severe peripheral vasoconstriction [35]. All of these factors have led cardiac surgeons to attempt to minimize catecholamines in lieu of non-catecholamine options.

### Vasopressin

Vasopressin is a synthetic form of AVP, also known as anti-diuretic hormone (ADH), with selective activity for the V1 receptor located on vascular smooth muscle. It causes smooth muscle contraction via a G-protein coupled receptor agonism, which stimulates a phosphatidylinositol-calcium signaling pathway and water reabsorption via aquaporin-2 water channels through an increase in cyclic AMP [36]. Vasopressin may also modulate the production of NO as well as potentiate the adrenergic response to stress [5]. ADH is synthesized in the pituitary gland and released in response to hypotension or increased osmolarity [37]. Vasopressin may be particularly attractive in vasoplegia due to the depletion that occurs during CPB. Argenziano et al. evaluated 145 post-CPB patients and found a significantly lower AVP level in those patients with vasoplegia (12.0 ± 6.6 vs. 29.3 ± 15.0, *P* = .004) [20]. Reasons for reduction in AVP during CPB are multifactorial, but may include the neurohumoral effects of elevated cardiac filling pressures pre-operatively, elevations in atrial natriuretic peptide (ANP), or autonomic dysregulation [20, 38, 39].

Like catecholamines, vasopressin has been evaluated primarily in patients with septic shock and has not shown a benefit in mortality [40, 41]. However, a recent meta-analysis found a significantly lower incidence of adverse events such as atrial fibrillation and need for renal replacement therapy [42]. As part of the aforementioned VANCS trial, Hajjar et al. evaluated vasopressin versus norepinephrine as first-line therapy in the treatment of vasoplegic syndrome post-cardiothoracic surgery. The composite outcome of mortality or severe complications was significantly lower in the vasopressin group, which was driven by a lower incidence of atrial fibrillation and acute renal failure [34]. The incidence of atrial fibrillation in the catecholamine group was greater than 80%, higher than a recently reported randomized trial comparing percutaneous coronary intervention to coronary artery bypass grafting (CABG) [43]. This may be due to higher catecholamine maximum ranges compared to typical practice patterns. Prior to VANCS, smaller trials have demonstrated an improvement in MAP and a decrease in catecholamine vasopressor requirements without an increase in adverse effects [20, 44]. Argenziano et al. showed that vasopressin administered to 40 post-CPB patients with vasoplegia increased MAP and reduced the requirement for catecholamine pressor agents in all patients [20]. Likewise, Morales et al. showed that in patients receiving ACE inhibitors preoperatively, vasopressin reduced the need for catecholamines after CPB as well as duration of vasopressor therapy [44].

Lower doses (0.04 units/min) of vasopressin may act to replete deficient stores of AVP in the post-operative period, but escalating doses of vasopressin are used frequently. Higher doses have questionable benefit and carry an increased risk of ischemia [45]. Despite this, doses higher than 0.06 units/min are often used in place of escalating catecholamines and may reflect the emerging belief in the benefit of de-catecholaminization.

### Ascorbic acid, thiamine, and corticosteroids

A combination of ascorbic acid (vitamin C), thiamine, and corticosteroids may mitigate vasoplegia via a number of mechanisms. Ascorbic acid is a cofactor for production of endogenous catecholamines, but is not synthesized by humans [46]. Its antioxidative properties may counteract excessive production of reactive oxygen species, which are known to cause decreased vascular tone and endothelial injury [47, 48]. .In animal models, intravenous ascorbic acid was shown to improve arteriolar responsiveness to vasoconstrictors and decrease microvascular permeability [49, 50]. However, when given at high doses, ascorbic acid may result in hyperoxaluria. Thiamine has been shown to decrease the conversion of ascorbic acid to oxalate preventing hyperoxaluria and also improves clearance of lactate by acting as a cofactor for metabolism of lactate by lactate dehydrogenase [51]. Glucocorticoids inhibit the arachidonic acid pathway, nuclear translocation of NF-kB transcription factor, synthesis of iNOS and COX2, and increase genetic expression of adrenergic receptors which have previously been downregulated [52–54]. They may also work synergistically with ascorbic acid to increase catecholamine synthesis, improve endothelial function, and increase vasopressor sensitivity [55–57].

This three-drug regimen was recently evaluated in a retrospective study of patients with sepsis or septic shock [58]. Marik et al. demonstrated a significant improvement in mortality as well as a rapid and significant reduction in vasopressor requirements in a retrospective cohort of patients receiving 6 g of ascorbic acid, 200 mg hydrocortisone, and 400 mg thiamine daily in divided doses. Large-scale evaluations of ascorbic acid, thiamine, and corticosteroids are currently ongoing. Evidence supporting the use of ascorbic acid in the setting of CPB is scant. A recent pilot trial evaluating intravenous vitamin C after cardiac surgery showed no statistically improved time to resolution of vasoplegia, norepinephrine dose, or ICU stay [59]. Extracorporeal circulation is known to reduce levels of ascorbic acid [60]. In a case series of three cardiac surgery patients, Wieruszewski et al. noted a reduction in vasopressor requirements in all three patients after the administration of ascorbic acid [51]. Two of the three patients did not require vasopressor support at 24 h.

Corticosteroids alone may help to re-establish blood pressure in vasoplegic syndrome, though studies have primarily focused on a septic shock population [61–65]. Experimental studies have shown restoration of vascular responsiveness to vasopressors, believed to be due to multiple pathways both genetic and non-genetic [11]. However, at this time, few clinical trials have evaluated the use of corticosteroids specifically for treatment of vasoplegic syndrome. Smaller studies have previously shown a decrease in inflammatory response associated with CPB [66]. More recently, two larger clinical trials demonstrated no benefit with the use of methylprednisolone or dexamethasone intraoperatively, but outcomes evaluated were not specific to blood pressure response or vasoplegic syndrome [67, 68]. The use of corticosteroids in the setting of cardiac surgery may be associated with delayed wound healing and poor glycemic control [6]. However, hydrocortisone 200 mg daily dose may be reasonable in patients requiring prolonged doses of vasopressors to address any concerns for adrenal insufficiency.

### Methylene blue

Methylene blue (MB) is indicated for use in the acute management of methemoglobinemia, where in low concentrations it facilitates the conversion of methemoglobin to hemoglobin. However, there is a growing body of evidence that suggests it may have a role in the management of vasoplegia following CPB, among other vasodilatory shock syndromes. MB has been shown to increase vascular smooth muscle tone [69]. Lenglet et al. proposed that the mechanism of action involves the inhibition of both eNOS and guanylate cyclase which work in tandem with sympathetic vasopressors to reduce vasodilation and improve hemodynamic stability [70]. However, the use of MB may cause hemolytic anemia in patients with a glucose-6-phosphate dehydrogenase (G6PD) deficiency, which is essential for the metabolism of the drug. Likewise, MB is a potent inhibitor of monoamine oxidase, and patients who are taking other serotonergic medications may be at risk of serotonin syndrome. Other notable side effects include interference with co-oximetry, with the potential to falsely lower apparent oxygen saturation due to the inhibition of light transmission by the blue dye. Importantly, Leyh et al. noted dose-dependent cardiac arrhythmias, coronary vasoconstriction, impaired gas exchange, and decreases in cardiac output, mesenteric, and renal blood flow in patients who receive doses of MB greater than 2 mg/kg [9]. Despite these risks, MB has been evaluated extensively for vasoplegic syndrome.

The dosing and administration of MB for post-CPB vasoplegia varies widely in the available literature, with some patients receiving the drug prior to CPB initiation, some during CPB, and yet others postoperatively. The preoperative administration of MB was formally evaluated by Ozal et al. in 2005 in a cohort of 100 high-risk patients undergoing CABG who were randomized to either MB 2 mg/kg given 1 h prior to surgery, or control [71]. SVR significantly improved, norepinephrine requirements were significantly reduced, and clinical signs of vasoplegia were less common in the MB group versus control. ICU/hospital length of stay was also reduced. Of note, this trial excluded patients with LVEF < 35%, a substantial limitation as this criterion is an independent risk factor for post-CPB vasoplegia development.

Intraoperative MB administration has been more extensively defined in the literature. Ribeiro et al. prospectively examined intraoperative MB use in a 60-patient cohort randomized to MB 2 mg/kg administered over 6 h or control [72]. The MB group had significantly higher diastolic blood pressures and SVR at 3 and 6 h respectively. They also noted lower TNF-alpha and NO levels post-CPB, suggesting reduced inflammation and vasodilation. Maslow et al. examined a 30-patient cohort taking ACE inhibitors randomized to MB 3 mg/kg given after initiation of CPB versus placebo. A significant rise in MAP and reduced phenylephrine use was noted in the MB group versus placebo, and lower lactate levels would seem to indicate a favorable effect on peripheral tissue perfusion. No significant difference in PaO_2_ was noted, indicating that MB did not impair gas exchange in the patients examined [73]. Most recently, Mehaffey et al. retrospectively examined 118 patients who received MB for vasoplegia in the setting of CPB and noted that mortality rates overall are high in patients who receive MB, but that early administration of the drug (given intraoperatively) demonstrated favorable outcomes in reduced mortality and incidence of renal failure when compared with late administration (post-operatively) [74]. Finally, Habib et al. evaluated MB use retrospectively in 28 patients matched to historical controls and found an improvement in mortality and time to discontinuation of all vasopressors in the MB group [75]. Importantly, no dose-finding studies have ever evaluated different doses of MB in patients with vasoplegia. The commonly utilized dose for shock of 2 mg/kg is extrapolated from methemoglobinemia treatment (one intravenous infusion of 1–2 mg/kg).

### Hydroxocobalamin

Hydroxocobalamin is indicated in the treatment of cyanide toxicity and is noted to have a side effect of increased blood pressure (CYANOKIT package insert (single 5-g vial), Columbia, MD: Meridian Medical Technologies, Inc.; 2017). The mechanism of hydroxocobalamin-induced blood pressure response remains unknown, but is believed to be related to the NO pathway [76]. Hydroxocobalamin is a potent direct inhibitor of NO as well as NO synthase [77, 78]. Additionally, hydroxocobalamin modifies innate hydrogen sulfide, an endothelial-bound endogenous vasodilator, increasing elimination [79]. Like MB, patients receiving hydroxocobalamin are at risk for certain side effects, including chromaturia, nausea, erythema, nephrolithiasis, lymphocytopenia, and infusion site reactions (CYANOKIT package insert (single 5-g vial), Columbia, MD: Meridian Medical Technologies, Inc.; 2017). Chromaturia may last several weeks and has the potential to interfere with hemodialysis machines, causing false blood leak alarms [79]. Importantly, hydroxocobalamin may also be associated with acute renal failure by virtue of increased risk of oxalate nephropathy [80].

Recently, case reports and series have demonstrated an increase in MAP in patients with vasoplegic syndrome when hydroxocobalamin is administered at a dose of 5 g over 15 min [81–85]. The largest cohort of patients published found a variety of responses when used in refractory cases [76]. Of those 33 patients, nine patients had no response, and the rest either had an adequate initial response, prolonged response, or had rebound hypotension within 2 h. Hydroxocobalamin was also evaluated in a case report of two patients with vasoplegic syndrome, in which a positive fluid balance was reversed after administration [86]. Barker et al. compared the effects of hydroxocobalamin to MB in 58 patients, 29 in each group, and found similar response in MAP, vasopressor requirements at 1 h, time to discontinuation of vasopressors, and length of stay, and a higher incidence of renal replacement therapy in patients receiving hydroxocobalamin compared to MB alone (PENDING PUBLICATION). However, a large number of these patients also received MB prior to hydroxocobalamin and were sicker in general. Similarly to MB, no dose-finding studies of hydroxocobalamin in the vasoplegia population have been completed. Dosing is extrapolated from the treatment of cyanide poisoning (5 g administered by IV infusion over 15 min × 1–2 doses).

Importantly, the data on inhibition of NO synthase, the proposed mechanism of action in both MB as well as hydroxocobalamin, is equivocal. The direct inhibitor of NO synthase G-methyl-L-arginine hydrochloride was shown to resolve shock in patients with severe sepsis [87]. Conversely, Lopez et al. showed that iNOS inhibition was associated with increased mortality in patients with septic shock [88]. Accordingly, NO synthase inhibition as a therapeutic goal should be pursued with caution.

### Angiotensin II

Angiotensin II is an endogenous peptide produced by the liver as angiotensinogen, and subsequently cleaved by renin in the kidney to angiotensin I and by lung endothelial-bound ACE to angiotensin II. The numerous effects of angiotensin II include direct arterial vasoconstriction by engagement of the AT-1 receptor on vascular smooth muscle, stimulation of aldosterone release, increased ADH secretion, and increase in sympathetic activity [89]. Potentiation of aldosterone and ADH result in sodium and water retention which increases intravascular volume and enhance blood pressure. Counter-regulatory effects are mediated by engagement of angiotensin II on the AT-2 receptor, which causes vasodilation as well as inotropy, in addition to the metabolism of angiotensin I to angiotensin 1–7, which is itself vasodilatory [90]. Angiotensin II in the setting of post-CPB vasoplegia is particularly attractive, as extra-corporeal circulation would be expected to bypass pulmonary circulation and thereby limit exposure of angiotensin I to ACE. A phase 3 clinical trial demonstrated a decrease in need for catecholamine vasopressors and improvement in MAP in septic patients receiving angiotensin II [91]. A small number of these patients (*n* = 19) diagnosed with vasoplegic syndrome post-cardiothoracic surgery were included in the trial. Of those patients, ten received angiotensin II with an adequate response in nine of the ten. Evans et al. described the first case of the use of synthetic human angiotensin II for vasoplegia following CPB, and a subsequent recent case report described successful down-titration of catecholamines with the use of angiotensin II in four patients with vasoplegia following CPB [92, 93].

#### Approach to treatment

Currently, there are no data supporting one non-catecholamine therapy over the others. A balanced approach in the management of vasoplegic syndrome may be optimal, allowing for lower doses of both catecholamine and non-catecholamine therapies so as to avoid the dangers of toxicity [94–96]. Current Society of Thoracic Surgeons (STS) guidelines refrain from addressing the treatment of vasoplegic syndrome, though there is a consensus statement recommending judicious use of epinephrine in post-surgical arrest [97]. Of relevance, the authors of this consensus statement argue against excessive epinephrine so as to avoid hypertension, a concept not totally unrelated to the idea of catecholamine sparing. Based on the best available evidence, vasopressin may be considered as a first-line non-catecholamine agent in combination with catecholamines. While evidence is equivocal regarding the blood pressure effect of vitamin C, thiamine, and steroids, these agents should be considered when two or more vasopressors are required to maintain adequate perfusion pressures, considering the potential benefit and low risk associated with these therapies. The initiation of additional therapies is appropriate when perfusion goals cannot be met with norepinephrine and vasopressin alone. Attempts have been made to protocolize the approach to treatment of vasoplegia [98]. Such algorithms highlight an emerging consensus regarding the dangers of excessive catecholamine use and feature lower doses of catecholamines as well as various non-catecholamine vasopressors. Whether this would lead to improved outcomes is to be determined. Because of the number of options available, protocols can become complicated, especially when including dose and titration recommendations, which anecdotally has been problematic vis-a-vis multiple titratable options. Importantly, no standard of care exists regarding the norepinephrine doses at which initiation of non-catecholamine therapy should begin. In a protocol by Ortoleva et al., non-catecholamine therapy is recommended to begin at norepinephrine doses of 0.5 μg/kg/min, which has been associated, at least in the distributive shock population, with an unacceptable level of mortality [95, 98].

**Fig. 2:**
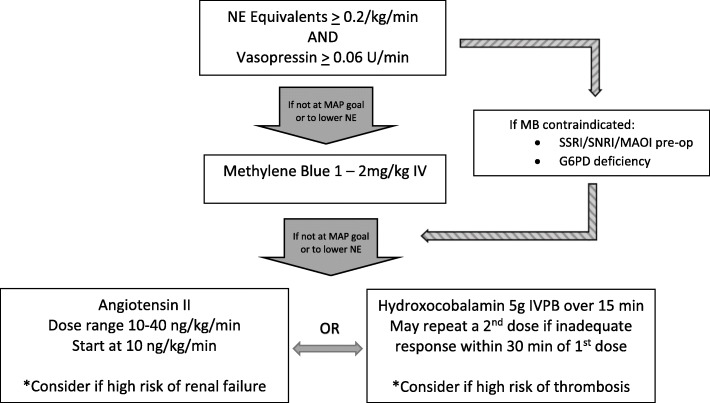
An approach to the treatment of vasoplegia. Non-catecholamine agents should be started at low doses, followed by non-catecholamine agents, including vasopressin and methylene blue. Use of hydroxocobalamin and/or angiotensin II should be considered with increasing doses of catecholamines. Clinical judgment should guide avoidance of certain agents if there is undue risk of side effects. All agents can be associated with intolerance, and discontinuation of offending agent(s) should be made accordingly

The authors’ approach to vasoplegia is presented as Fig. 2. Non-catecholamine agents should be started at lower doses of catecholamines (0.1 mcg/kg/min), with the first-line non-catecholamine agent being vasopressin, followed by methylene blue. Thereafter, hydroxocobalamin and/or angiotensin II should be used once catecholamine doses reach 0.2 μg/kg/min. Care should be taken to identify potential risk factors for intolerance or adverse reaction, and avoidance or discontinuation of the offending agent should be made accordingly.

Additionally, the response to each agent should be evaluated, with discontinuation of any agent which yields an ineffective response (such as lack of an increase in MAP or a concomitant up-titration of other agents). Finally, attention should be paid to titration of adjustable agents, so as to avoid excessive or prolonged use. A titration table is presented as Table 2.

**Table 2:** Vasopressor titration^a^

NE equivalent^b^ (μg/kg/min)	Vasopressin (U/min)	Methylene blue^c^ (mg/kg)	Angiotensin II (ng/kg/min)^d,e^	Hydroxycobalamin^f^ (g)
If you have just titrated NE to:	Make sure vaso is:	Administer	And titrate ang II to:	Administer
< 0.05	< 0.07	No	Ang II off^c^	No
0.05–0.1	< 0.07	Yes	10^c^	Yes
0.1–0.15	< 0.07	Yes	20	Yes
0.15–0.20	< 0.07	Yes	30	Yes
> 0.20	< 0.07	Yes	40	Yes

^a^Titration driven by NE dosing, based on MAP goals

^b^NE equivalent doses represented in Table 1

^c^2 mg/kg IVP over 5 min or as IVPB over 20–60 min

^d^Ang II maximum dose is 40 ng/kg/min

^e^Always initiate Ang II at 10 ng/kg/min. In patients who are hyper responders or extremely hemodynamically dependent on Ang II, consider titrating down Ang II to 5 ng/kg/min before titrating off

^f^5 g infused over 15 min

## Conclusion

Vasoplegic syndrome may occur in up to half of all patients undergoing cardiothoracic surgery, with predisposing patient-specific risk factors combined with inflammatory response to CPB as precipitating causes. NO is believed to play a large role in refractory vasodilation and thus is a potential target for therapies. While catecholamines are considered first-line therapy in vasoplegic syndrome, non-catecholamine agents may be considered early or in place of catecholamines by virtue of their improved safety profile with regard to cardiac toxicity. Moreover, these agents may be associated with improved outcomes such as reduced kidney injury. Data is largely circumstantial and hypothesis-generating, but there is emerging consensus that catecholamine sparing may lead to improved clinical outcomes after CPB. In general, the treatment of vasoplegic syndrome should be rational and balanced, with the judicious use of catecholamine and non-catecholamine agents alike. Further efforts are required to validate the various protocols, including the one presented here, for effectiveness in patient-centered outcomes.

## Data Availability

Not applicable.
